# Identifying barriers to the acceptability and adoption of ambulatory blood pressure monitoring and proposed strategies in Bangladesh, Pakistan, and Sri Lanka: a qualitative study

**DOI:** 10.1186/s12913-026-14107-y

**Published:** 2026-02-03

**Authors:** Anqi Zhu, Sungwon Yoon, Aysha Almas, Aliya Naheed, H. Asita de Silva, Chamini Kanatiwela de Silva, Lathika Athauda, Nantu Chakma, Muhammad Shahid Khan, Noshin Farzana, Tazeen H. Jafar

**Affiliations:** 1 Program in Health Services Research and Population Health, Duke-NUS Medical School, Singapore, Singapore; 2 Centre for Population Health Research and Implementation, SingHealth Regional Health System, Singapore, Singapore; 3 Internal Medicine Department of Medicine, Aga Khan University, Karachi, Pakistan; 4 Nutrition Research Division, Non-Communicable Diseases, International Centre for Diarrhoeal Disease Research (icddr, b), Dhaka, Bangladesh; 5 Department of Pharmacology, Faculty of Medicine, University of Kelaniya, Kelaniya, Sri Lanka; 6 RemediumOne, Colombo, Sri Lanka; 7 Department of Public Health, Faculty of Medicine, University of Kelaniya, Kelaniya, Sri Lanka; 8 Duke Global Health Institute, Durham, NC USA

**Keywords:** Qualitative research, Hypertension, Ambulatory blood pressure monitoring, South Asia, Acceptability

## Abstract

**Introduction:**

In South Asia, hypertension is a leading modifiable risk factor for premature death. Ambulatory blood pressure monitoring (ABPM) is an internationally recommended method for hypertension diagnosis, but remains underutilized in South Asia, with little evidence on users’ perspectives. This study aims to identify barriers and proposed strategies related to the acceptability and adoption of ABPM for hypertension management from the perspectives of patients with hypertension and healthcare professionals in Bangladesh, Pakistan, and Sri Lanka.

**Methods:**

Across government and private facilities in Bangladesh, Pakistan, and Sri Lanka, we performed semi-structured interviews with 35 patients with hypertension who had previous experience of using ABPM. We also interviewed 29 healthcare professionals involved in hypertension management who had knowledge on ABPM use, including 11 technicians, 15 specialist physicians, 2 primary care physicians, and 1 administrative head. Guided by a combined framework of the Theoretical Framework of Acceptability and the Consolidated Framework of Implementation Research, we used framework analysis to identify barriers and proposed strategies.

**Results:**

While patients with hypertension and healthcare professionals generally supported expanding ABPM use across all three countries, social stigma around ABPM emerged as a common cultural barrier for patients in South Asia. Other key barriers included low public awareness, limited public availability and insurance coverage, lack of organizational alignment and readiness, and patient burden and inconvenience. Participants proposed strategies to address these barriers, including public awareness campaigns to promote and normalize ABPM use, expanding public access through government and donor support, improving ABPM integration by standardizing clinical pathways and strengthening the workforce, and developing more user-friendly ABPM device.

**Conclusion:**

In resource-constrained South Asian settings, our findings highlight the need for public education and stronger government support to improve ABPM adoption and acceptability. We provide actionable and context-sensitive insights to inform interventions aimed at reducing social stigma and expanding equitable ABPM access, and to support local guidelines and policies for improving hypertension management through acceptable approaches.

**Supplementary Information:**

The online version contains supplementary material available at 10.1186/s12913-026-14107-y.

## Introduction

Elevated blood pressure (BP) is a predominant modifiable risk factor for cardiovascular disease (CVD) and premature deaths in South Asia [1–3]. In 2019, the prevalence of hypertension in South Asia was estimated to be 31.1%, contributing to 16.5% of all-cause deaths [1, 4]. South Asian individuals are known to experience higher vascular risk compared to other ethnic groups, with more adverse effects of elevated BP on stroke [5, 6]. Early and accurate diagnosis of hypertension is thus essential in South Asia, where hypertension-related morbidity and mortality are expected to surge in the coming decades [7, 8]. 

The current hypertension diagnostic approach in South Asia still primarily relies on the conventional clinic BP measurement, which is poorly reproducible and often leads to over- or under-diagnosis [9]. Out-of-office BP measurements, such as home blood pressure measurement (HBPM), are therefore increasingly recommended for hypertension diagnosis and management [10]. However, HBPM cannot access nocturnal BP or dipping patterns [10]. Moreover, the reliability of HBPM were affected by lack of user training, limited access to validated devices, and low adherence, which were found common in South Asia [11]. A more reliable out-of-office alternative is ambulatory blood pressure monitoring (ABPM) that can automatically measure BP at fixed time intervals during an individual’s daily activities. Additionally, ambulatory BP measurements are superior to clinic BP in predicting target organ damage, cardiovascular events, and overall mortality [12]. Established hypertension guidelines, including those in South Asia, have consistently advocated for the use of ABPM to confirm hypertension diagnosis and to help with therapeutic decision-making during follow-up [9, 10, 13, 14]. However, similar to other low-and middle-income countries (LMIC), the use of ABPM remained limited in South Asia [15–17]. 

While system-level resource constraints pose challenges to adopting ABPM in South Asia [18], understanding its acceptability among patients with hypertension and healthcare professionals is a necessary first step before wider adoption [19, 20]. In addition to discomfort and inconvenience [21–23], previous studies in Western countries have found that stigma, especially among people of South Asian origin, was a significant barrier to patient acceptability for ABPM [24]. However, few studies have explored the acceptability of ABPM in South Asia.

Therefore, the objective of this study was to identify barriers to the acceptability and adoption of ABPM for hypertension management among patients with hypertension and healthcare professionals in Bangladesh, Pakistan, and Sri Lanka, and to explore potential strategies to address these barriers. This report focuses mainly on the identified barriers and participant-proposed strategies to support ABPM adoption. Our findings will spur the discussion in this region and other LMIC on appraising acceptable and effective BP measuring approaches, tailoring them to local conditions, and informing progressive local guidelines and policies to assess and prevent vascular risk.

## Methods

### Study design

Between December 2023 and January 2025, we conducted a qualitative study using semi-structured interviews to explore the lived experiences and perceptions of patients with hypertension (“patients”) and healthcare professionals across a few selected government and private facilities in Bangladesh, Pakistan, and Sri Lanka. Participants were primarily approached at tertiary care facilities, with some recruited from primary and secondary levels. In Bangladesh, patients who underwent ABPM as part of the COBRA-BPS trial (NCT02657746, registered 14/01/2016) were approached [25]. Based on estimates from previous reviews, we aimed for a minimum of seven patients and seven healthcare professionals per country, and ended recruitment when data saturation was reached through a consensus between the Singapore and local teams [26, 27]. The ethics review committees or institutional reviews boards at each participating institution approved the study. The study is reported in line with the Consolidated Criteria for Reporting Qualitative Research (COREQ) [28]. 

### Study settings

Located in South Asia, Bangladesh, Pakistan, and Sri Lanka are classified as low-middle-income countries with large and growing populations [29–31]. In 2019, the estimated age-standardized prevalence of hypertension among adults was 28.8% in Bangladesh, 43.2% in Pakistan, and 35.6% in Sri Lanka [32]. All three countries have mixed healthcare systems comprising government and private facilities. Bangladesh and Pakistan healthcare deliveries are dominated by private sectors, particularly in urban areas [33, 34]. In contrast, Sri Lanka provides strong, government-funded universal health coverage for all citizens, with private sector use largely based on patient preference and affordability [35]. Details of ABPM service provision, implementation, and maintenance observed in this study are summarized in Table S1 (Additional File 1).

### Theoretical frameworks

We combined components from the Theoretical Framework of Acceptability (TFA) and Consolidated Framework for Implementation Research (CFIR) for data collection and analysis (see Additional File 1: Figure S1). The TFA provides a structured approach to evaluate acceptability as an individual’s perceived appropriateness of the healthcare intervention [36]. The CFIR, a widely used implementation framework, helps to explain “why” intervention may or may not be successful when implemented across various settings, including resourced-constrained ones [37, 38]. Since combining theoretical frameworks was common for studies with multifaceted objectives [39, 40], this study used the TFA and CFIR together to effectively guide acceptability research and intervention evaluation in a resource-constrained setting by elucidating how barriers affected acceptability.

### Study participants

Eligible participants included patients aged 40 years or older with a diagnosis of hypertension and prior exposure to ABPM, as well as healthcare professionals actively involved in hypertension management who knew what ABPM is used for and how it works (see Additional file 1: Table S2 for detailed criteria). We first approached potential participants to obtain informed consent, then assessed their eligibility using a questionnaire tailored to each group (see Additional file 2 for questionnaires). In Pakistan and Sri Lanka, we recruited patients from cardiology units or equivalent facilities providing ABPM services, either face-to-face when they returned the device or by phone using records of ABPM users within the past 12 months. Due to limited access to organizations providing ABPM service, in Bangladesh, patients who underwent ABPM as part of the COBRA-BPS trial were approached and interviewed in their communities [25]. We showed these patients a photo of ABPM device and excluded those unable to recall the experience to reduce bias. All healthcare professionals were recruited from departments involved in hypertension management and/or ABPM provision in person or by phone.

We recruited eligible participants using purposive sampling to ensure diversity in age, sex, comorbidities (patients), and professional roles (healthcare professionals) [41]. Healthcare professionals were categorized into technicians (healthcare professionals responsible for ABPM device application), specialist physicians (physicians working in or training for specialties such as cardiology or nephrology), primary care physicians (physicians providing general or primary care), and administrative heads (individuals overseeing clinical services) based on their own role descriptions. These terms were used throughout the study for consistency, although alternative titles were used by some participants. We aimed to recruit at least seven patients and seven healthcare professionals in each country based on prior studies [26]. Recruitment ended in each country once the Singapore and the local teams jointly reviewed the data and agreed that data saturation had been reached, and no new insights had emerged from the last three interviews that would expand our analysis in new directions [26, 27]. The participant flow chart showing recruitment conditions is shown in Figure S2 (Additional file 1).

### Data collection

Data were collected by research teams trained in qualitative research in the three countries with backgrounds in medicine, public health, or social science. To ensure consistency and data quality, we also conducted two remote workshops on study design, interview techniques, data management, and transcription, followed by a third workshop on NVivo 14 for data analysis.

Sociodemographic data were collected via the same questionnaire used for eligibility screening. We then conducted semi-structured interviews in the participant’s preferred language, including Bangla, English, Sindhi, Sinhala, Tamil, and Urdu. Both questionnaire and interview guides were initially developed in English and later translated into local languages by each respective local team. Interview guides were developed based on our combinational framework of TFA and CFIR, tailored by participant group, and refined based on insights from early interviews (see Additional file 3 for interview guides). Interviews took place in a quiet room at the hospital or at a location chosen by the participants, such as their office or home. All interviews lasted 30 to 60 min and were audio-recorded with consent. In each country, data collectors completed debriefing notes for the first three interviews per participant group to refine their interviewing techniques.

### Data transcription and analysis

All interviews were either transcribed directly into English (Pakistan) or first transcribed in the original language and subsequently translated into English (Bangladesh, Sri Lanka). Each transcript was reviewed by the corresponding coordinator (CKdS, NC, and MSK), who rechecked the recordings to ensure accuracy. All transcripts and questionnaire data were de-identified prior to analysis.

Guided by the combinational framework of TFA and CFIR, we used framework analysis to identify patterns in the data through both deductive and inductive coding [42]. Deductive coding was based on a priori codes from the combinational framework, while inductive coding could identify emerging themes from participant’s experience. Line-by-line coding was conducted in NVivo 14 software [43]. 

The principal analyst (AZ) developed an initial codebook in consultation with PI (THJ) and Co-I (SY) based on the first three transcripts per participant group in Pakistan. This codebook was iteratively refined through internal feedback and then shared with all teams. In each country, the first three transcripts per participant group were independently coded by AZ and the local analysts (LA, NC, and MSK), in consultation with the respective site PIs (AA, AN, and HAdS). To ensure cultural and linguistic accuracy, two additional transcripts per participant group were independently analyzed by local analysts in the original language (or by listening to recordings in Pakistan) and by AZ using English translations. Coding discrepancies were discussed during biweekly meetings and resolved through consensus. Each team adjusted the codebook during the process to capture emerging context-specific themes focusing on barriers and potential solutions for wider ABPM adoption. The remaining transcripts were then divided between AZ and the local analysts for coding based on the updated codebook, with additional codes added as needed (see Additional file 4 for codebook). The PI and site PIs oversaw the analysis process and were consulted in cases where discrepancies emerged.

## Results

We interviewed a total of 64 participants across Bangladesh (*n* = 18), Pakistan (*n* = 24), and Sri Lanka (*n* = 22), including 35 patients and 29 healthcare professionals (Table 1, Additional file 1: Table S2). The average age of the patients was 54.2 years (SD = 9.7) and 17 (48.6%) were female. Among healthcare professionals, 11 (37.9%) were technicians, 15 (51.7%) were specialist physicians, 2 (6.9%) were primary care physicians, and 1 (3.4%) was an administrative head. The average age of the healthcare professionals was 39.9 years (SD = 9.0) and 14 (48.3%) were female.

**Table 1:** Sociodemographic characteristics of patients with hypertension and healthcare professionals

Participant Characteristics	Bangladesh (*N* = 18)	Pakistan (*N* = 24)	Sri Lanka (*N* = 22)	Overall (*N* = 64)
**Patients with Hypertension**	(*N* = 11)	(*N* = 12)	(*N* = 12)	(*N* = 35)
Age in year, mean (SD)	60.1 (7.7)	54.5 (10.7)	48.4 (6.9)	54.2 (9.7)
Female, n (%)	5 (45.5%)	5 (41.7%)	7 (58.3%)	17 (48.6%)
Residency status, n (%)				
Urban	0 (0.0%)	11 (91.7%)	3 (25.0%)	14 (40.0%)
Rural	11 (100.0%)	1 (8.3%)	9 (75.0%)	21 (60.0%)
Highest level of education^*^, n (%)				
Primary education	7 (63.6%)	0 (0.0%)	0 (0.0%)	7 (20.6%)
Secondary education	3 (27.3%)	1 (8.3%)	5 (41.7%)	9 (25.7%)
Tertiary education	1 (9.1%)	11 (91.7%)	7 (58.3%)	19 (54.3%)
Employment status, n (%)				
Employed or actively working	1 (9.1%)	8 (66.7%)	9 (75.0%)	18 (51.4%)
Unemployed or not currently working	7 (63.6%)	4 (33.3%)	1 (8.3%)	12 (34.3%)
Retired	3 (27.3%)	0 (0.0%)	2 (16.7%)	5 (14.3%)
**Healthcare Professionals**	(*N* = 7)	(*N* = 12)	(*N* = 10)	(*N* = 29)
Age in year, mean (SD)	35.1 (4.5)	36.5 (10.1)	47.3 (4.7)	39.9 (9.0)
Female, n (%)	3 (42.9%)	9 (75.0%)	2 (20.0%)	14 (48.3%)
Practice setting, n (%)				
Private	3 (42.9%)	12 (100.0%)	2 (20.0%)	17 (58.6%)
Government	4 (57.1%)	0 (0.0%)	0 (0.0%)	4 (13.8%)
Dual practice (government & private)	0 (0.0%)	0 (0.0%)	8 (80.0%)	8 (27.6%)
Professional role^†^, n (%)				
Technicians	2 (28.6%)	5 (41.7%)	4 (40.0%)	11 (37.9%)
Specialist physicians	3 (42.9%)	6 (50.0%)	6 (60.0%)	15 (51.7%)
Primary care physicians	2 (28.6%)	0 (0.0%)	0 (0.0%)	2 (6.9%)
Administrative heads	0 (0.0%)	1 (8.3%)	0 (0.0%)	1 (3.4%)
Direct experience with prescribing or applying ABPM, n (%)	2 (28.6%)	8 (66.7%)	9 (90.0%)	19 (65.5%)

^*^Education levels were categorized into three groups: primary education (no formal education or completed primary school), secondary education (completed lower or upper secondary school), and tertiary education (completed trade school, vocational training, college, or university). ^[44] †^Professional roles were categorized into four groups: technicians (healthcare professionals responsible for ABPM device application), specialist physicians (physicians working in or training for specialties such as cardiology or nephrology), primary care physicians (physicians providing general or primary care), and administrative heads (individuals overseeing clinical services). Abbreviations: ABPM, ambulatory blood pressure monitor; SD, standard deviation

**Fig. 1:**
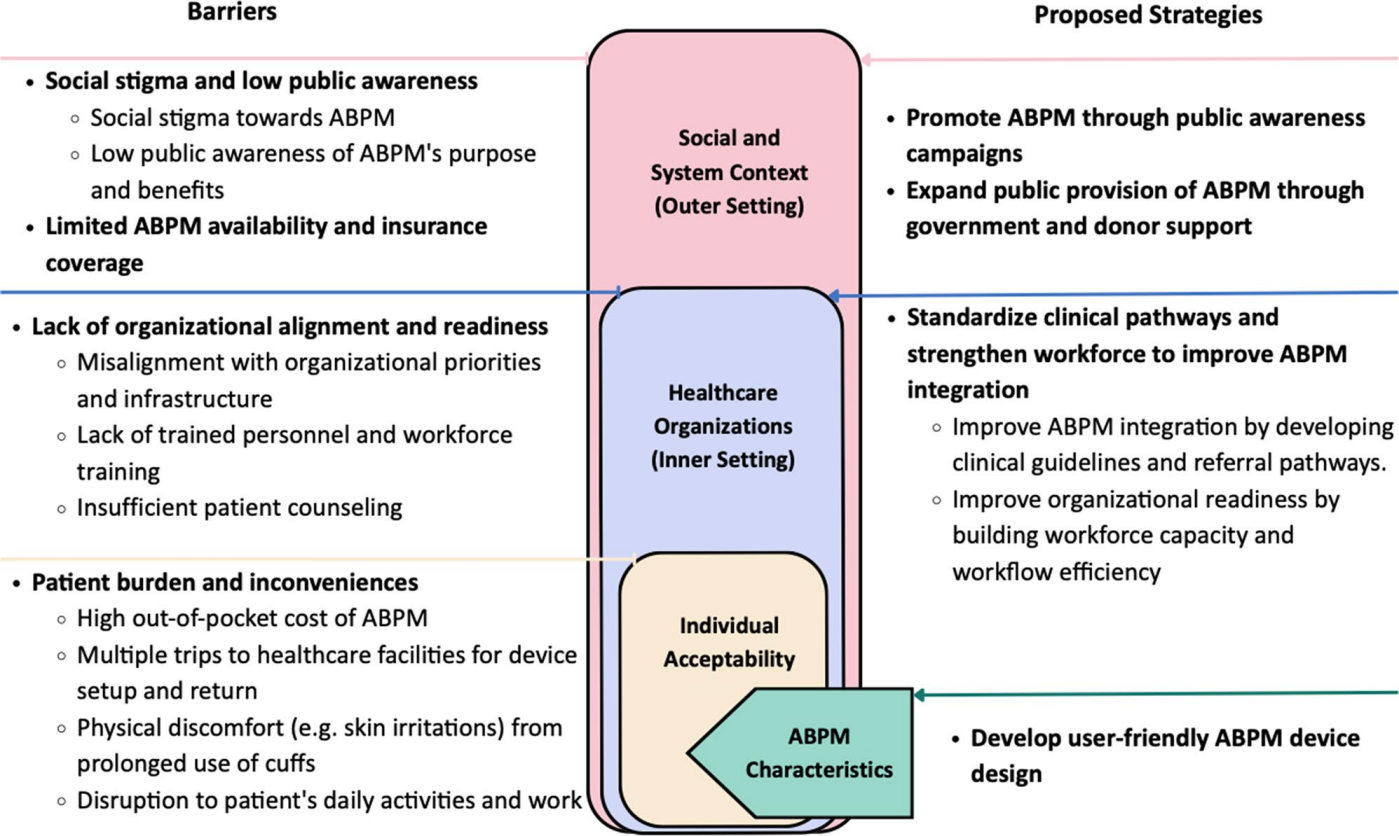
Barriers and proposed strategies for ABPM acceptability and adoption in Bangladesh, Pakistan, and Sri Lanka. This figure summarizes key barriers and proposed strategies for the acceptability and adoption of ABPM among patients with hypertension and healthcare professionals based on the combinational framework of theoretical framework of acceptability and the consolidated framework of implementation research in Bangladesh, Pakistan, and Sri Lanka. Abbreviations: ABPM, ambulatory blood pressure monitoring

Figure 1 provides an overview of the key themes, with Tables 2 and 3 presenting illustrative quotes related to barriers and strategies proposed by participants to support ABPM adoption and acceptability. The results below are organized thematically, starting with the key barriers followed by corresponding strategies. Given the cross-cutting nature of the themes across CFIR domains, findings are presented as an integrated thematic synthesis rather than categorized separately by domain

**Table 2:** Participant quotes illustrating barriers for ABPM acceptability and adoption

Theme	Constructs	Participant Quotes
Social stigma and low public awareness	Social stigma	• “People did not know what kind of device I was using. … I did not feel good that they were looking at me like I had a very dangerous disease. I was very disturbed.” (PK-PT-04, male, 63 years old, Pakistan)
	Low public awareness	• “When I was using [the ABPM], everybody at my office came and observed it. … Nobody knew about it or heard of it, even though many of them have hypertension.” (SL-PT-06, female, 50 years old, Sri Lanka)
Limited public availability and insurance coverage	--	• “In the private facility, if I request [ABPM], it happens in a few days. But in the government facility … months later you get a date. There are only a few machines, and those are occupied all the time.” (SL-HP-08, male, specialist physician, Sri Lanka)
Lack of organizational alignment and readiness	Misalignment with organizational priorities and infrastructure	• “We have been requesting ABPM for the government facility for a long time. We haven’t received it yet, as this is not considered a priority.” (SL-HP-04, male, technician, Sri Lanka) • “It’s difficult to administer [ABPM] at the Upazila Health Complex level due to shortage of manpower and heavy patient load. And we don’t have that machine and expertise.” (BD-HP-05, male, specialist physician, Bangladesh)
	Lack of trained personnel and workforce training	• “They don’t do the workshops, or it’s very minimal, [and] we don’t have free internet in the ward to check [the guidelines].” (SL-HP-02, male, specialist physician, Sri Lanka) • “I’ve never used [ABPM] and there’s no proper training for it, so I’m not confident.” (PK-HP-11, female, technician, Pakistan)
	Insufficient patient counseling	• “[The ABPM result] was written on my [prescription] paper, but it was not explained to me.” (BD-PT-05, male, 56 years old, Bangladesh)
Patient burden and inconveniences	Financial, physical, and logistical burden for patients	• “[Considering the cost of ABPM, unless] the doctor deems it necessary… Otherwise, it’ll be a decision made by my family.” (PK-PT-03, female, 48 years old, Pakistan) • “It’d be better if we could do [ABPM] from a nearby place… It felt far with travelling, [and] the inconvenience was with the distance.” (SL-PT-12, male, 59 years old, Sri Lanka) • “It was very hot, and I was itching a lot. [The ABPM test] was very difficult.” (PK-PT-11, male, 48 years old, Pakistan)
	Disruption to patients’ daily activities and work	• “[The ABPM device] was a big, bulging thing attached to my abdomen. … It’s heavy, and it made working a bit difficult.” (SL-PT-11, female, 51 years old, Sri Lanka)

Abbreviations: ABPM, ambulatory blood pressure monitoring; HP, healthcare professionals; PT, patients with hypertension

**Table 3:** Participant quotes illustrating proposed strategies for ABPM acceptability and adoption

Theme	Constructs	Participant Quotes
Promote ABPM through public education campaigns	--	• “It’s like advertising to [the patients]. You can inform them through the mass media: Newspaper, [television] channels, or radio, arrange an awareness program and educate them about the [ABPM’s] significance.” (BD-HP-02, male, specialist physician, Bangladesh)
Expand public provision of ABPM through government and donor support	--	• “In the private facility, we must pay around 10,000–15,000 rupees to get this [ABPM] test done. So, having it available in the government facility is a big thing, a meritorious thing for the poor person.” (SL-PT-08, male, 40 years old, Sri Lanka) • “The acceptability and usage [of ABPM] will be more if it’s readily available. For example, there’re NGO and welfare associations that provide ventilators [in] our government setups.” (PK-HP-10, female, specialist physician, Pakistan)
Standardize clinical pathways and strengthen workforce to improve ABPM integration	Improve ABPM integration by developing clinical guidelines and referral pathways	• “Others should also recommend [ABPM] other than cardiologists. … Internists, general physicians, and family physicians should also be informed [so] that BP of more patients could be assessed properly.” (PK-HP-04, female, specialist physician, Pakistan)
	Improve organizational readiness by building workforce capacity and workflow efficiency	• “We can set some criteria [for ABPM usage] and, for [supporting its use in our organization], we can arrange symposiums and seminars.” (BD-HP-01, female, specialist physician, Bangladesh) • “If [the doctor] took the report and discussed [with us] why the pressure got raised, it’d be better. … We could know whether it’s a normal or abnormal case.” (SL-PT-07, male, 42 years old, Sri Lanka)
Develop user-friendly ABPM device design	--	• “When I left home, I took [the ABPM] with me. … It’d be good if it were [lightweight], slim, beautiful, and had its own nice packaging.” (PK-PT-01, female, 41 years old, Pakistan)

Abbreviations: ABPM, ambulatory blood pressure monitoring; HP, healthcare professionals; NGO, non-governmental organizations; PT, patients with hypertension

### Barriers to ABPM adoption

We identified four key barriers to ABPM adoption and acceptability, including social stigma and low public awareness, limited public availability and insurance coverage, lack of organizational alignment and readiness, and patient burden and inconvenience (Fig. 1; Table 2**)**. Since most differences in responses reflected resources availability rather than country-specific patterns, we report findings across all three countries unless noted.

#### Social stigma and low public awareness

##### Social stigma

Patients from all countries reported experiencing social stigma when wearing ABPM in public. The bulky device and the visible wires connecting the cuff and the main unit often attracted unwanted attention, including stares, questions, and sometimes judgmental looks from both strangers and people from their daily lives. This visibility made some patients feel embarrassed or demeaned, as they believed they were perceived as “weak” or “sick”. Several patients also described staying indoors, avoiding social events, or attempting to conceal the device to avoid having to “explain to everyone”. Healthcare professionals across all sites confirmed that social stigma created a psychosocial barrier for patients, making some “reluctant” to undergo ABPM when recommended. They were concerned that this has limited ABPM’s broader acceptance and raised challenges in ensuring respectful care for all patients.

##### Low public awareness

Low public awareness was consistently identified as a major barrier in all three countries. Both patients and healthcare professionals believed ABPM was more accurate than clinic or home BP measurements and provided an “actual diagnosis” to guide treatment. However, they mentioned that most people in their community, even those with hypertension, were unaware of ABPM test. The lack of accessible information in local languages contributed to patients’ confusion, and their uncertainty about the value of the test. Healthcare professionals noted that such knowledge gap was closely linked to stigma, and deterred patients from using ABPM.

#### Limited ABPM availability and insurance coverage

Another commonly noted barrier was the limited availability of ABPM service, especially in the government facilities, and the lack of insurance coverage. Most patients and healthcare professionals perceived that ABPM was underutilized across all three countries and limited mostly to private facilities, potentially due to a shortage of ABPM devices and limited public financial support.

The shortage of ABPM devices, particularly in resource-constrained settings including rural and many urban facilities-constrained settings, significantly limited their public availability. Healthcare professionals noted that organizations in these settings usually relied on unstable procurement sources, such as donations or personal purchases. However, donated ABPM devices often lacked vendor or organizational support for maintenance, leaving healthcare professionals to replace them only after they broke down. Devices purchased personally by healthcare professionals were tied to individuals rather than organizations, raising concerns about their long-term sustainability. These procurement challenges further exacerbated the already limited availability of devices. Some healthcare professionals also linked this limited availability to the absence of government initiatives, noting that without policies, government facilities lacked both funding and motivation to invest in ABPM.

Both patients and healthcare professionals also expressed concerns about the lack of public financial support and price regulation for ABPM services. Given the limited public health insurance in Bangladesh and the lack of ABPM coverage in Pakistan, most individuals in both countries rely on out-of-pocket payments. While a few patients in Pakistan reported receiving reimbursement through employment-based insurance, this created unequal access based on employment status. In Sri Lanka, the lack of price regulation was also reported to cause significant cost variation for ABPM across private facilities, further complicating its accessibility.

#### Lack of organizational alignment and readiness

##### Misalignment with organizational priorities and infrastructure

Some healthcare professionals reported that ABPM was not well aligned with their organization’s priorities or operational workflows, limiting its routine use. Under the tight budget constraints, other equipment seen as in higher demands or more profitable were prioritized over ABPM. In primary healthcare settings, some healthcare professionals were also hesitant to adopt ABPM as it would add “hassle” to their already busy workflows. The overloaded healthcare settings left little capacity for them to take on additional clinical responsibilities related to it. They thus felt ABPM was better suited for specialized or tertiary care settings, where more time and resources could be dedicated for patient counseling and device setup. Instead, these healthcare professionals in primary healthcare settings continue to prioritize clinic BP measurement, which they believed better aligned with their organization’s purpose in screening BP for the general population.

The lack of organizational policies and infrastructure to support device management might further contribute to this misalignment. Healthcare professionals reported challenges such as lack of BP cuff sizes for patients in special needs, and a limited number of BP cuffs making proper sanitization between uses difficult. Sweat could cause cuffs to become smelly, leading some patients to refuse ABPM testing due to fears of infection risks. In government hospitals in Sri Lanka, patients were typically asked to stay overnight during ABPM testing, which conflicted with its purpose of capturing daily BP patterns. Healthcare professionals there expressed concerns about patients not returning or mishandling devices, potentially due to the absence of formal deposit policy.

##### Lack of trained personnel and workforce training

The shortage of trained personnel for ABPM was another frequently mentioned barrier limiting its adoption within organizations. Many healthcare professionals noted that their colleagues were unfamiliar with ABPM. This was usually compounded by healthcare professionals’ limited access to formal, structured training. Many healthcare professionals in rural areas or among junior roles relied on self-directed learning through textbooks, internet resources, international conferences, or vendor demonstrations. In settings without official access to up-to-date guidelines, healthcare professionals also depended on peer support network to stay informed and managed ABPM services.

Such limited access to workforce training and guidelines posed a major barrier to healthcare professional adoption of ABPM. This might lead to variation in clinical practices related to ABPM, including how and when healthcare professionals referred patients for testing. Healthcare professionals with limited hands-on training were also less confident in using and interpreting ABPM. Concerns such as unexpected device malfunctions and doubts about whether patients would adhere to instructions further contributed to their hesitation in recommending or applying ABPM in routine practice.

##### Insufficient patient counseling

Patients across countries reported receiving little or no explanation during counseling about how the ABPM device worked or how to interpret the results, reflecting gaps in organizational readiness. This lack of counseling left them uncertain about how their ABPM readings informed clinical decisions regarding hypertension status or treatment. These patients were therefore hesitant to undergo ABPM test again due to their limited understanding of its purpose and value. In Pakistan, a lack of guidance on how the device would be attached (holding the main unit with wires secured to clothing using paper tapes) caused further uncertainty, with some patients arriving in unsuitable clothing.

#### Patient burden and inconvenience

##### Financial, logistical, and physical burden for patients

Many patients, whether paying out-of-pocket or receiving coverage through trial (Bangladesh) or insurances, perceived the current cost of ABPM to be high and unfair. Although ABPM was generally viewed as a reliable method for hypertension diagnosis and management, patients expressed concerns that the high cost could create an immediate financial burden, turning it into a “family decision” to proceed with the test. Many patients considered themselves “fortunate” to afford ABPM, but argued that the high cost placed it out of reach for most others. Healthcare professionals shared similar concerns, noting their moral dilemma when recommending ABPM to financially struggling patients. Some attempted to direct patients to cheaper facilities when available. In Bangladesh, patients were generally less willing to pay for future ABPM use compared to those in Pakistan and Sri Lanka, considering it unsuitable for routine use and only worth paying for unless absolutely necessary.

Additionally, some patients expressed frustrations with the need of multiple trips to the organizations for ABPM setup and device return. According to the healthcare professionals, some organizations charged extra fees if the device was not returned the following day. Therefore, patients from remote areas often had to travel long distances or stay near the organizations for several days.

ABPM could also introduce physical discomfort from prolonged cuff use. Many patients in our study reported some forms of “tolerable” discomfort, ranging from itchiness to mild pain. Healthcare professionals, reflecting on their previous experience, also recalled a few rare cases of more severe reactions such as rashes and allergies. These issues were exacerbated by the oftentimes warm, humid climate and patients’ inability to bathe during the test, leading a few patients to hesitate about taking ABPM again.

##### Disruption to patient’s daily activities and work

Most patients reported that ABPM interfered with their daily activities, such as sleep, exercise, and religious practices like prayers. Some minimized outdoor activities during the test, partly due of concerns about the wires detaching or malfunctioning, and partly to isolate themselves out of fear of social stigma. The extent of work disruption varied depending on job requirements. Patients with office-based or light housekeeping responsibilities reported less or no disruption. In contrast, those with physically demanding jobs often had to take leave during the test, even though some of them relied on daily wages and thus lost income.

### Proposed strategies to increase ABPM adoption

In this section, we identified four key strategies proposed by our participants relating to the identified barriers, including promoting ABPM through public awareness campaigns, expanding public access through government and donor support, improving ABPM integration by standardizing clinical pathways and strengthening the workforce, and developing more user-friendly ABPM device (Fig. 1; Table 3**)**.

#### Promote ABPM through public awareness campaigns

To reduce social stigma and raise awareness, many patients and healthcare professionals called for public education campaigns to increase community knowledge of hypertension and the benefits of ABPM in its management. Suggested approaches included community events and health promotion materials in local languages disseminated via websites, radios, televisions, and other mass media platforms. Some healthcare professionals also advocated these efforts as a means to normalize ABPM use and reduce social stigma.

#### Expand public provision of ABPM through government and donor support

Government was widely seen as the key player in addressing barriers to ABPM adoption in resource-constrained settings. A common suggestion was greater government support to make it more affordable. Proposed strategies included government-facilitated bulk import agreements to reduce device costs for healthcare organizations, and public subsidies to enable free or low-cost ABPM in government facilities. Although supports from non-governmental organizations (NGO) and personal donors were reported in Bangladesh and Sri Lanka, it remained insufficient to meet the system-wide demand. Some healthcare professionals proposed greater collaboration with international NGO to complement government funding, particularly in regions where public resources were constrained.

In Sri Lanka, current universal health policy provided partial coverage for ABPM in government hospitals, with patients responsible for buying batteries. While many patients and healthcare professionals viewed this policy as a “strong support”, they still called for full coverage to further improve the accessibility of ABPM. Greater investment in devices for government facilities was also suggested, as their oftentimes limited devices caused long waiting times for the patients.

Several healthcare professionals also proposed national policies requiring hospitals, particularly the tertiary-care facilities, to provide ABPM testing for high-risk individuals to increase its accessibility. To reach patients living in the remote areas, healthcare professionals suggested increasing the availability of ABPM devices in underserved regions and employing community health workers to deliver and retrieve the devices directly from doorstep.

#### Standardize clinical pathways and strengthen workforce to improve ABPM integration

##### Improve ABPM integration by developing clinical guidelines and referral pathways

To better integrate ABPM into clinical workflows, healthcare professionals recommended organizations developing clear clinical guidelines and referral pathways. They mentioned that developing clear clinical guidelines for ABPM, especially with local validation of its diagnostic thresholds and treatment recommendations, would help both cardiologists and general physicians to more readily identify and refer appropriate patients needing ABPM testing. These guidelines should also include policies for devices management and maintenance to ensured “dependable” ABPM results. In settings where ABPM was not available, establishing referral pathways and adopting electronic health record systems to facilitate patient access to external ABPM services were proposed.

##### Improve organizational readiness by Building workforce capacity and workflow efficiency

To improve organizational readiness for ABPM adoption, healthcare professionals advocated for organizations to offer regular training workshops and educational materials to strengthen their capacity and confidence in using and interpreting ABPM.

In resource-constrained settings, task-sharing was also suggested to improve workflow efficiency, delegating ABPM-related counseling and testing responsibilities to nurses or technicians. Patients also proposed take-home instructional pamphlets as a complement to counseling, with key points about device use and care during the test.

#### Develop user-friendly ABPM device design

Many patients and healthcare professionals expressed their desire for ABPM devices with improved designs that could reduce physical discomfort and social stigma, therefore increasing acceptability. They recommended making ABPM device lighter and smaller so that it would be easier and more comfortable to wear throughout daily activities. Some patients also wondered whether a tubeless or cuffless design might be possible, as such innovations would greatly improve convenience. These suggestions, along with proposed alternative ways to carry the device such as discreet harnesses or necklaces worn underneath clothing, also reflected a desire to reduce the device’s visibility in public, which may help alleviate the social stigma and embarrassment associated with wearing ABPM.

## Discussion

In this qualitative study of patients with hypertension and healthcare professionals in Bangladesh, Pakistan, and Sri Lanka, social stigma around wearing the device in public emerged as a common cultural barrier. Low public awareness, limited availability and insurance coverage, lack of organizational alignment and readiness, patient burden and inconvenience were also identified as key barriers. To address these barriers, participants proposed four main strategies: promoting ABPM and normalizing its use through public awareness campaigns, expanding public access through government and donor support, improving ABPM integration by standardizing clinical pathways and strengthening the workforce, and developing a more user-friendly ABPM device. Of note, wider adoption of ABPM as part of routine clinical services was supported by most patients and healthcare professionals in all three countries. These findings can inform culturally sensitive strategies to improve the acceptability and expand the adoption of ABPM in resource-constrained South Asian settings.

Our study adds to current evidence by demonstrating that social stigma surrounding ABPM use was also present among patients in South Asian countries, leading some to isolate themselves or forgo future testing. While prior studies suggested that people of South Asian origin experienced greater “embarrassment” wearing ABPM compared to their white British counterparts, no study has explored this issue in South Asia [21, 24]. In our study, stigma of ABPM appeared to stem from both low public awareness, which led to misconceptions about the device, and cultural pressures to conceal health conditions among the South Asian communities [45, 46]. These were supported by qualitative studies on non-communicable diseases, where South Asian individuals hid their insulin devices or refused antihypertensive treatments altogether to avoid being identified as a “patient” [45–47].

Our findings are consistent with literature from developed countries in identifying key organizational and individual barriers to ABPM [21–23, 48–50], which may be further compounded by contextual challenges in South Asia, such as inadequate infrastructure and insufficient workforce training. In addition, hot, humid climate in South Asia reportedly increased the risk of excessive sweating and skin irritation from the APBM arm cuff, especially for those working outdoors. The large proportion of the local population relying on daily wages also meant that taking time off work to complete the test imposed a greater financial burden [51]. While we explored cross-country differences during analysis, the perceptions of our patients and healthcare professionals on barriers to ABPM adoption were largely consistent across settings. However, we observed some country-specific nuances in how ABPM was perceived, with patients in Bangladesh recounting less sufficient explanation of the test. They also appeared less willing to pay for future ABPM use and more uncertain about its reliability compared to those in Pakistan and Sri Lanka.

Our participants proposed discreetly wearing the device under clothing or adopting less noticeable device design to reduce stigma around ABPM. Recent innovations in cuffless BP monitors, such as wristbands, smartwatches, or rings, may support these needs as well as mitigate discomfort associated with humid environments [52, 53]. Despite their growing development, many of these novel wearable devices remain costly and face ongoing challenges related to validation standards and integration into clinical guidelines [54, 55]. Targeted public health campaigns were also proposed to improve awareness and reduce stigma of ABPM among South Asian populations. Based on prior experience [56], community presentations and televised discussions to address myths and misconceptions surrounding hypertension and ABPM may help normalize its use as a routine part of hypertension management, especially among older adults.

The major strength of this study is adding novel insights as the first study that explored ABPM acceptability and adoption across Bangladesh, Pakistan, and Sri Lanka. By integrating perspectives from both patients and healthcare professionals and adopting a combined framework of TFA and CFIR, we systematically identified barriers and proposed strategies to inform future ABPM implementation in resource-constrained settings.

This study also had a few limitations. First, inconsistencies in types of healthcare settings and participant profiles included across countries prevented direct cross-country comparisons. Nevertheless, participants’ views on ABPM were largely consistent between countries, suggesting shared perspectives across South Asian contexts. Second, our study population might not fully represent the broader population receiving ABPM in the three countries. We restricted recruitment to patients aged 40 and above to be consistent with the trial participants in Bangladesh, despite ABPM at times being prescribed to younger patients with elevated BP. Third, some patients paid out-of-pocket for ABPM while those in Bangladesh received it free in our previous trial, introducing potential selection and recall bias. These factors may affect the comparability and generalizability of our findings.

High blood pressure is a well-established leading risk factor for cardiovascular morbidity and mortality [10]. ABPM detects masked hypertension missed by clinic measurements and offers distinctive information on nighttime BP, a stronger predictor of overall and CVD mortality than clinic BP [10, 12]. Given the elevated hypertension-related risk in South Asian populations [5, 6], integrating ABPM for hypertension diagnosis may also be cost-efficient [57–61]. At the same time, the larger challenge in hypertension care remains low awareness, treatment, and control in South Asia [62, 63]. Many of our participants therefore emphasized a cautious approach to ABPM expansion to prevent “abusing” it given limited healthcare resources. Interventions promoting ABPM adoption could be integrated with broader hypertension education and lifestyle programs, where ABPM could complement HBPM for hypertension diagnosis and management. Future studies should assess when and for which patient group ABPM would be a cost-effective option, to ensure equity is maintained in any scale-up across this region.

## Conclusions

In Bangladesh, Pakistan, and Sri Lanka, we conducted one of the first qualitative studies to identify barriers to ABPM acceptability and adoption among patients with hypertension and healthcare professionals. While patients and healthcare professionals generally supported expanding ABPM use, our findings add to current evidence by identifying social stigma around wearing ABPM in public as a common cultural barrier for South Asian population. Other key barriers included low public awareness, limited public availability and insurance coverage, lack of organizational alignment and readiness, and patient burden and inconvenience. Public education to promote ABPM and normalize its use, stronger government support to improve ABPM adoption and acceptability, and innovative smaller wearable validated devices are needed to improve uptake of ABPM in South Asia. Our findings provide actionable and context-sensitive insights to inform interventions aimed at expanding equitable ABPM access in resource-constrained South Asian context, and to support local guidelines and policies for achieving more accurate hypertension diagnosis and improved cardiovascular risk stratification.

## Supplementary Information

Below is the link to the electronic supplementary material.

Supplementary Material 1

Supplementary Material 2

Supplementary Material 3

Supplementary Material 4

Supplementary Material 5

## Data Availability

Deidentified data analyzed during this study are available from the corresponding author upon reasonable request.

## Abbreviations

ABPM: Ambulatory Blood Pressure Monitoring
BP: Blood Pressure
CFIR: Consolidated Framework for Implementation Research
CVD: Cardiovascular Disease
HBPM: Home Blood Pressure Monitoring
LMIC: Low- and Middle-Income Countries
NGO: Non-Governmental Organizations
TFA: Theoretical Framework of Acceptability
