# Supplementary material for: Identifying barriers to the acceptability and adoption of ambulatory blood pressure monitoring and proposed strategies in Bangladesh, Pakistan, and Sri Lanka: a qualitative study
Source: BMC Health Serv Res. 2026 Feb 3;26:237. doi: 10.1186/s12913-026-14107-y (PMC12903567; doi:10.1186/s12913-026-14107-y)
Supplement: Supplementary file 1 — Supplementary Material 1 [file 12913_2026_14107_MOESM1_ESM.docx]

**Supplementary File 1 – Supplementary Results**

**Identifying Barriers to the Acceptability and Adoption of Ambulatory Blood Pressure Monitoring and Proposed Strategies in Bangladesh, Pakistan, and Sri Lanka: A Qualitative Study**

Anqi Zhu ^a^, Sungwon Yoon ^a, b^, Aysha Almas ^c*^, Aliya Naheed ^d*^, H. Asita de Silva ^e*^, Chamini Kanatiwela de Silva ^f^, Lathika Athauda ^g^, Nantu Chakma ^d^, Muhammad Shahid Khan ^c^, Noshin Farzana ^d^, Tazeen H. Jafar ^a, h^

^a^Program in Health Services Research and Population Health, Duke-NUS Medical School, Singapore, Singapore

^b^Centre for Population Health Research and Implementation, SingHealth Regional Health System, Singapore, Singapore

^c^Internal Medicine Department of Medicine, Aga Khan University, Pakistan.

^d^Non-Communicable Diseases, Nutrition Research Division, International Centre for Diarrhoeal Disease Research (icddr, b), Dhaka, Bangladesh.

^e^Department of Pharmacology, Faculty of Medicine, University of Kelaniya, Kelaniya, Sri Lanka.

^f^RemediumOne, Colombo, Sri Lanka.

^g^Department of Public Health, Faculty of Medicine, University of Kelaniya, Kelaniya, Sri Lanka.

^h^Duke Global Health Institute, Durham, NC, USA

*****These authors contributed equally to the study.

**Corresponding Author:** Professor Tazeen H. Jafar, MD MPH. Program in Health Services Research and Population Health, Duke-NUS Medical School, Singapore; Duke Global Health Institute, Durham, USA. Email: [tazeen.jafar@duke-nus.edu.sg](mailto:tazeen.jafar@duke-nus.edu.sg); ORCID: <https://orcid.org/0000-0001-7454-8376>

# SUPPLEMENTARY TABLES

## Table S1 Contextual Overview of ABPM Service Provision in Selected Healthcare Settings Across Bangladesh, Pakistan, and Sri Lanka

|  | **Bangladesh** | **Pakistan** | **Sri Lanka** |
| --- | --- | --- | --- |
| ***Provision of ABPM service*** | | | |
| Study settings | - Government tertiary hospitals. - Government secondary facilities. - Government primary facilities. - Private tertiary hospitals. | - Private tertiary hospital. | - Government tertiary hospitals. - Private tertiary hospitals. |
| Availability of ABPM devices | - Limited to a few private tertiary hospitals. | - ABPM devices adequate for current demand. - Also available in some other private tertiary hospitals. | - Limited to private tertiary hospitals and a few governmental hospitals. - Usually, one to two devices per hospital. |
| Sources of ABPM devices | - Purchased by individual private hospitals | - Purchased by individual private hospitals based on patient demand. | - Procured primarily through individual purchases and donations from individuals or NGO. - Some purchased by individual private hospitals. |
| ***Implementation of ABPM*** | | | |
| Costs | - Cost relatively high at private hospitals ( ~ 24.6 USD per test)^*^ | - Cost relatively high at private hospitals ( ~ 64.8 USD per test)^*^ - Other private hospitals offered at similar or lower costs. | - In the private hospitals, costs varied a lot (range from ~13.6 to 68.0 USD per test)^*^ - In the government hospitals, costs were subsidized (~2.0 USD per test) |
| Primary patients’ groups | - Resistant hypertension: Patients whose BP remains uncontrolled despite medication | - Complex hypertension cases: patients with comorbid conditions requiring accurate BP monitoring. - Resistant hypertension | - General hypertension diagnosis, particularly for younger patients with potential hypertension. - Resistant hypertension. |
| Patient experience | - Patients can take devices home. No advance payment needed. | - Patients can take devices home with a deposit. | - In the private hospital, patients can take devices home with a deposit. - In the government hospitals, patients must stay in-hospital during testing to prevent device loss. |
| ***Maintenance of ABPM*** | | | |
| Training and support | - Lack of formal training programs and practical experience for healthcare professionals on ABPM in most settings. - Vendors provided training workshop. | - Structured training programs including ABPM application at the private hospital. | - Lack of formal training programs on ABPM; reliance on peer support networks, internet, or informal training. |
| Technical support and repairs | - Devices could be repaired by vendors. | - Devices maintained through a mix of in-hospital repairs and manufacturer support. | - Limited repair support; broken devices typically need replacing. |

*All local costs were converted to USD using exchange rates as of January 2025: 1 BDT = 0.0082 USD, 1 PKR = 0.0036 USD, and 1 LKR = 0.0034 USD.

Abbreviations: ABPM, ambulatory blood pressure monitor; BDT, Bangladeshi Taka; BP, blood pressure; LKR, Sri Lanka Rupee; NGO, non-governmental organization; PKR, Pakistan Rupee; USD, United States dollar.

## Table S2 Participant recruitment criteria

|  | **Patients with Hypertension** | **Healthcare Professionals** |
| --- | --- | --- |
| **Inclusion Criteria** | - Patients with a diagnosis of clinic hypertension (clinic SBP ≥ 140 mmHg or DBP ≥ 90 mmHg on two or more prior visits, physician-diagnosed hypertension, or on antihypertensive medication). - Are of age 40 years or older. - Underwent clinic BP measurements at least twice during the last 12 months. - Underwent ABPM test at least once during the last 12 months and is able to recall the experience.^*^ | - Technicians, specialist physicians, and primary care physicians that:   - Are involved in hypertension management in South Asia   - Demonstrate understanding of ABPM, including its functionality and integration into patient care workflow.   - Prescribed or administered clinic BP measurements weekly during the last 3 months. - Administrative heads that demonstrate understanding of ABPM, including its functionality and integration into patient care workflow. |
| **Exclusion criteria** | - Any major debilitating disease or mental illness that precludes the validity of informed consent. |  |

^*^In Bangladesh, due to limited access to organizations providing ABPM, we recruited patient participants from the prior COBRA-BPS trial (NCT02657746), which provided free ABPM to patients aged 40 years or older with clinic hypertension from April 2016 to March 2019.^1^ During recruitment, a photo of ABPM device was shown to examine participants’ recall of their prior experience.

Abbreviations: ABPM, ambulatory blood pressure monitoring; BP, blood pressure; DBP, diastolic blood pressure; SBP, systolic blood pressure.

## Table S3 Characteristics of Patients with Hypertension and Healthcare Professionals

| **Participant Characteristics** | **Bangladesh  (N = 18)** | **Pakistan  (N = 24)** | **Sri Lanka  (N = 22)** | **Overall  (N = 64)** |
| --- | --- | --- | --- | --- |
| **Patients with Hypertension** | (N = 11) | (N = 12) | (N = 12) | (N = 35) |
| History of hypertension, n (%) |  |  |  |  |
| Less than five years | 2 (18.2%) | 8 (66.7%) | 8 (66.7%) | 16 (45.7%) |
| Five to ten years | 3 (27.3%) | 2 (16.7%) | 2 (16.7%) | 7 (20.0%) |
| More than ten years | 6 (54.5%) | 2 (16.7%) | 2 (16.7%) | 12 (34.3%) |
| Taking antihypertensive medications, n (%) | 11 (100.0%) | 9 (75.0%) | 12 (100.0%) | 32 (91.4%) |
| With comorbidity, n (%) | 6 (54.5%) | 6 (50.0%) | 6 (50.0%) | 17 (48.6%) |
| **Healthcare Professionals** | (N = 7) | (N = 12) | (N = 10) | (N = 29) |
| Frequency of ABPM usage, n (%) |  |  |  |  |
| Weekly | 2 (28.6%) | 4 (33.3%) | 5 (50.0%) | 11 (37.9%) |
| Monthly | 0 (0.0%) | 2 (16.7%) | 3 (30.0%) | 5 (17.2%) |
| Less than monthly | 5 (71.4%) | 6 (50.0%) | 2 (20.0%) | 13 (44.8%) |

Abbreviations: ABPM, ambulatory blood pressure monitoring.

# SUPPLEMENTARY FIGURES

Figure S1 Combinational framework of TFA and CFIR Domains Applied to ABPM Acceptability and Adoption in South Asia. The figure shows the combined theoretical framework used to guide our exploration of barriers to the acceptability and adoption of ABPM, as well as strategies proposed to address them. This framework integrates constructs from the TFA and selected domains from the CFIR. TFA was applied to explore individual-level acceptability, while CFIR offered a broader lens to explore factors at the healthcare organizational and social levels shaping adoption. The CFIR domains “Implementation process” and “Characteristics of individuals” were excluded, because the former was not relevant to our study aims and the latter overlapped conceptually with TFA constructs. Abbreviations: ABPM, ambulatory blood pressure monitoring; CFIR, consolidated framework of implementation research; TFA, the theoretical framework of acceptability.

## Figure S2 Participant Flowchart. This flowchart summarizes the participant recruitment process in Bangladesh, Pakistan, and Sri Lanka.

References**:**

1. Jafar TH, Gandhi M, de Silva HA, et al. A Community-Based Intervention for Managing Hypertension in Rural South Asia. *N Engl J Med* 2020;382(8):717-26. doi: 10.1056/NEJMoa1911965.
