# Supplementary material for: Identifying barriers to the acceptability and adoption of ambulatory blood pressure monitoring and proposed strategies in Bangladesh, Pakistan, and Sri Lanka: a qualitative study
Source: BMC Health Serv Res. 2026 Feb 3;26:237. doi: 10.1186/s12913-026-14107-y (PMC12903567; doi:10.1186/s12913-026-14107-y)
Supplement: Supplementary file 2 — Supplementary Material 2 [file 12913_2026_14107_MOESM2_ESM.docx]

**Supplementary File 2 – Questionnaires**

**Questionnaire for Patients with Hypertension**

**Introduction:** Thank you for agreeing to participate in the study. Now, I am going to ask you a few questions. There are no right or wrong answers to the questions, and you do not have to respond to any questions that you feel uncomfortable answering. Please be assured that your identity and the information given by you will be kept strictly confidential and that only group data will be reported. Please feel free to call the study team at Tel:___________ if you need any clarification on this study.

| Name of interviewer: |  |
| --- | --- |
| Participant’s ID: |  |

| 1. | Country | Bangladesh | 🗆 1 |  | 7. | Healthcare setting |  | |
| --- | --- | --- | --- | --- | --- | --- | --- | --- |
|  |  | Pakistan | 🗆 2 |  | 8. | Date of interview (dd/mm/yyyy) |  | |
|  |  | Sri Lanka | 🗆 3 |  | 9. | Language of interview | Urdu | 🗆 1 |
| 2. | Residence | Rural | 🗆 1 |  |  |  | Sinhala | 🗆 2 |
|  |  | Urban | 🗆 2 |  |  |  | Sindhi | 🗆 3 |
| 3. | Age (years) |  | |  |  |  | Tamil | 🗆 4 |
| 4. | Gender |  | |  |  | | Bengali | 🗆 5 |
| 5. | Mobile Ph # |  | |  | Other: ____________ | |  | 🗆 6 |
| 6. | Residence Ph # |  | |  |  |  |  |  |

| High Blood Pressure | | | |
| --- | --- | --- | --- |
| A1 | Has your blood pressure ever been checked in the past 12 months? | | |
|  | 1 |  | Yes |
|  | 2 |  | No |
| A2 | Has a health care provider ever told you that you have high blood pressure also called hypertension (other than during pregnancy)? | | |
|  | 1 |  | Yes |
|  | 2 |  | No |
| A3 | Have you ever been prescribed medicines by a healthcare provider for your high blood pressure? | | |
|  | 1 |  | Yes |
|  | 2 |  | No |
| A4 | How long have you known about your high blood pressure? | | |
|  | 1 | ______________ year(s) | |
| Ambulatory Blood Pressure Monitoring (ABPM) Experience | | | |
| A5 | Have you used the ABPM device to measure your blood pressure in the past 12 months? [Please refer to the photos of ABPM devices] | | |
|  | 1 |  | Yes |
|  | 2 |  | No (Not Eligible) |
|  | 3 |  | Don't know/ can’t remember (Not Eligible) |
| Socioeconomic Status | | | |
| A6 | What is your highest level of education? | | |
|  | 1 |  | No formal or primary school education only |
|  | 2 |  | Completed secondary school education |
|  | 3 |  | Completed trade or vocational school, college, or university education |
| A7 | What is your current employment status? | | |
|  | 1 |  | Employed or actively working |
|  | 2 |  | Retired |
|  | 3 |  | Unemployed or not currently working |
| Comorbidities | | | |
| A8 | Have you ever had a heart attack? | | |
|  | 1 |  | Yes |
|  | 2 |  | No |
|  | 3 |  | Don't know/ can’t remember |
| A9 | Has a health care provider ever told you that you have heart disease? | | |
|  | 1 |  | Yes |
|  | 2 |  | No |
|  | 3 |  | Don’t know |
| A10 | Has a health care provider ever told you that you have diabetes? | | |
|  | 1 |  | Yes |
|  | 2 |  | No |
|  | 3 |  | Don’t know |
| A11 | Have you ever had a stroke? | | |
|  | 1 |  | Yes |
|  | 2 |  | Never |
|  | 3 |  | Don’t know |

**Questionnaire for Healthcare Professionals**

**Introduction:** Thank you for agreeing to participate in the study. Please take a few moments to answer the following questions. There are no right or wrong answers to the questions, and you do not have to respond to any questions that you feel uncomfortable answering. Please be assured that your identity and the information given by you will be kept strictly confidential and that only group data will be reported. Please feel free to call the study team at Tel: ___________ if you need any clarification on this study.

| Name of interviewer: |  |
| --- | --- |
| Participant’s ID: |  |

| 1. | Country | Bangladesh | 🗆 1 |  | 6. | Healthcare setting |  | |
| --- | --- | --- | --- | --- | --- | --- | --- | --- |
|  |  | Pakistan | 🗆 2 |  | 7. | Date of interview (dd/mm/yyyy) |  | |
|  |  | Sri Lanka | 🗆 3 |  | 8. | Language of interview | Urdu | 🗆 1 |
| 2. | Age (years) |  | |  |  |  | Sinhala | 🗆 2 |
| 3. | Gender |  | |  |  |  | Sindhi | 🗆 3 |
| 4. | Mobile Ph # |  | |  |  |  | Tamil | 🗆 4 |
| 5. | Office Ph # |  | |  |  |  | Bengali | 🗆 5 |
|  |  |  | |  | Others: _______________ | |  | 🗆 6 |

| A1 | Are you currently practicing in a private healthcare setting? | | |
| --- | --- | --- | --- |
|  | 1 |  | Yes |
|  | 2 |  | No |
| A2 | What is your role in the healthcare setting? | | |
|  | 1 |  | Physician |
|  | 2 |  | Nurse |
|  | 3 |  | Administrator |
| A3 | Have you ever used the Ambulatory Blood Pressure Monitoring (ABPM) to monitor your patients’ blood pressure before? | | |
|  | 1 |  | Yes |
|  | 2 |  | No (Skip to A5) |
| A4 | How often do you use using the ABPM to monitor your patients’ blood pressure? | | |
|  | 1 |  | At least once a week |
|  | 2 |  | At least once a month |
|  | 3 |  | Less than once a month |
| A5 | Do you know what ABPM is used for and how it works? | | |
|  | 1 |  | Yes |
|  | 2 |  | Don't know/ can’t remember (Not Eligible) |
