# Supplementary material for: Identifying barriers to the acceptability and adoption of ambulatory blood pressure monitoring and proposed strategies in Bangladesh, Pakistan, and Sri Lanka: a qualitative study
Source: BMC Health Serv Res. 2026 Feb 3;26:237. doi: 10.1186/s12913-026-14107-y (PMC12903567; doi:10.1186/s12913-026-14107-y)
Supplement: Supplementary file 3 — Supplementary Material 3 [file 12913_2026_14107_MOESM3_ESM.docx]

**Supplementary File 3 – Interview Guides**

**Interview Guide for Patients with Hypertension**

Thank you for agreeing to take part in this study. Today, we are hoping to learn about your experience and acceptability of using the ambulatory blood pressure monitoring (ABPM). Before we begin, I’d like to explain a few things about the study and how the interview will work.

Firstly, this interview will take around 30-60 minutes. The conversation will be recorded and transcribed, but no confidential information will be mentioned. As a reminder, your participation in this study is voluntary. You may choose to not answer some of the questions or end the interview at any time. If you are unsure about anything we ask, please feel free to ask for clarification or ask us to repeat the question.

Secondly, we understand that there may be some confusion or misunderstanding about the ABPM device, particularly if you have experience using other approaches to measure blood pressure. Therefore, I would like to provide you with some information and show you some pictures of the device we will be discussing today. Ambulatory blood pressure monitoring, or ABPM, is a medical device that is used to automatically measure your blood pressure multiple times over a 24-hour period. Here are some photos of what the device looks like [show photos].

Before we begin, do you have any questions about what I have just explained? [answer questions]

May I start recording the interview? (After gaining permission, switch on the recorder) Ok, thanks for letting us record this.

**Opening questions**

1. To start with, please tell me about your high blood pressure. How has it affected your health and daily life?

*Probes:*

1. *What have you been doing to control your blood pressure? For example, are you currently taking any medications or receiving any treatments?*
2. Do you measure your blood pressure regularly? Why or why not?

*Probes:*

1. *When and how did you have your blood pressure measured last time? For example, did you measure it at home or go to a healthcare professional to have it checked?*
2. *What do you think are the benefits of measuring blood pressure regularly?*

**Questions on Experienced Acceptability**

1. Please share with me your experience in using the ambulatory blood pressure monitoring (ABPM) to measure your blood pressure. What made you decide to use it?

*Probes:*

- 1. *How many times did you wear the ABPM in the last 12 months? Do you have any plans to wear it again in the future? Why or why not?*
  2. *Did you experience any side-effects, discomforts, and inconveniences caused by wearing the ambulatory blood pressure monitor for 24 hours? For example, did you notice any skin irritation or pain in your arm from the cuff? If so, can you describe them? (Adverse effects)*
  3. *How did you feel about wearing the ABPM during your sleep? For example, did it wake you up at any point during the night, or did you have any trouble falling asleep because of it?*

1. What did you like or dislike about using ABPM? *(****Affective attitude****)*
2. Did you have any concerns or worries about using ABPM to measure your blood pressure? If so, can you tell me what you were worried about? *(****Ethicality****)*
3. How well do you think you understood how the ABPM device worked? (**Intervention Coherence**)

*Probes:*

- 1. *Did you receive any instructions on how to use ABPM? If so, were they clear and easy to follow?*

1. How confident were you in using ABPM? (**Self-efficacy**)

*Probes:*

- 1. *Did you have any concerns or doubts about using it correctly?*

1. How convinced were you that ABPM was effective in measuring your blood pressure correctly? *(****Perceived Effectiveness****)*

*Probes:*

- 1. *How did ABPM compare to your current blood pressure measuring approach in terms of accuracy and ease of use? (Relative advantage)*

1. How much effort did you feel was required on your part to use ABPM? (**Burden**)

*Probes:*

1. *Did you have to pay for ABPM yourself or was it provided to you for free? If you had to pay for it, did the cost of ABPM create any significant financial challenges for you? (Cost)*
2. Did you have to give up anything in order to use ABPM to measure your blood pressure? (**Opportunity cost**)

*Probes:*

1. *Did it have any impact on your daily routine or activities? For example, how did you feel about revisiting the clinic or facility to return the ABPM device? Did using ABPM affect your daily prayers? If so, how did it affect your religious practices? (time)*
2. *Did you experience any unexpected cost related to using ABPM? If so, please describe in more detail. (money)*

**Questions on using ABPM in the clinic/facility.**

1. Based on your experience of using ABPM, what are your thoughts on using it in your doctor’s clinic or facility?

*Probes:*

- 1. *How well do you think ABPM suits you and other hypertensive patients? (Patient needs and resources)*
  2. *Did the cost of ABPM affect your decision to use or continue using ABPM to measure your blood pressure?*

1. What changes or adjustments do you think should be made to make using ABPM better fit your needs?

*Probes:*

- 1. *Do you think you will use ABPM again if it suits you better?*
  2. *Do you think there are any groups of patients who might benefit from using ABPM? Why and why not?*

1. What kind of support do you think is needed to make things easier for patients like yourself to use ABPM?

*Probes:*

- 1. *If your doctor recommended you use ABPM, what else would you want to know about it before deciding whether to take it?*
  2. *Would you tell me more about what changes your doctor’s clinic or facility needs to make?*

1. Do you have anything else that you would like to share on this topic?

That's the end of our interview. Thank you for taking your time with me today. [Turn off the recorder]

**Interview Guide for Healthcare Professionals**

Thank you for agreeing to take part in this study. Today, we are hoping to learn about your acceptability of using ambulatory blood pressure monitoring (ABPM) for blood pressure monitoring in the clinic/facility and its potential influencing factors. Before we begin, I’d like to explain a few things about the study and how the interview will work.

Firstly, this interview will take around 30-60 minutes. The conversation will be recorded and transcribed, but no confidential information will be mentioned. As a reminder, your participation in this study is voluntary. You may choose to not answer some of the questions or end the interview at any time. If you are unsure about anything we ask, please feel free to ask for clarification or ask us to repeat the question.

Secondly, we understand that there may be some confusion or misunderstanding about the device, particularly if you have experience using other approaches for blood pressure monitoring. Therefore, I would like to provide you with some information and show you some pictures of the device we will be discussing today. Ambulatory blood pressure monitoring, or ABPM, is a medical device that is used to measure your blood pressure at regular intervals over a 24-hour period. Here are some photos of what the device looks like [show photos].

Before we begin, do you have any questions about what I have just explained? [answer questions]

May I start recording the interview? (After gaining permission, switch on the recorder) Ok, thanks for letting us record this.

**Opening questions**

1. To start with, would you tell me something about your current position at [organization]?

*Probes:*

- 1. *What is your current position/title?*
  2. *How long have you worked here?*

1. In your position, what are your roles and responsibilities in relation to patient care for patients with hypertension?

*Probes:*

1. *Are you in charge of blood pressure measurements?*
   1. *(If so) how do you measure their blood pressure?*
   2. *(If not) how are you involved in the blood pressure measuring process?*
2. *Are you aware of any guidelines that you and your colleagues used during this process?*

**Questions on Experienced Acceptability**

1. Please share with me your experiences of performing ambulatory blood pressure monitoring (ABPM) in your practices.

*Probes:*

- 1. *Would you walk me through the steps involved in performing ABPM and what was your role in the process?*

1. What are your thoughts on ABPM?

*Probes:*

- 1. *What did you like or dislike about performing ABPM on your patients with hypertension? (****Affective attitude****)*
  2. *In your opinion, does performing ABPM align with your values and beliefs as a healthcare professionals? Why and why not? (****Ethicality****)*
  3. *Compared to other blood pressure measuring methods, what features or characteristics did ABPM have that stood out to you? (Relative advantage)*

1. How convinced were you about the effectiveness of ABPM? (**Perceived Effectiveness**)

*Probes:*

- 1. *How did ABPM compare to other methods of blood pressure measurement in terms of accuracy and patient compliance?*
  2. *How did your patients respond to using ABPM? Did they find it easy or difficult to use? Tell me more about what they said.*

1. How confident were you to successfully perform ABPM for your patients? (**Self-efficacy**)

*Probes:*

- 1. *Were there any particular aspects of performing ABPM that made you feel less confident?*
  2. *Have you received any training on the proper use of this device? If so, could you describe the training you received? If not, why not?*

1. On a scale of 1-10, how would you rate your understanding of how ABPM works? Would you elaborate on this? (**Intervention Coherence**)

*Probes:*

- 1. *What kind of information or materials about using ABPM were made available to you (access to knowledge & information)?*

1. How much effort did you take to perform ABPM? (**Burden**)

*Probes:*

- 1. *How complicated was performing ABPM? (****Complexity****)*
  2. *What were your thoughts on the possible adverse effects for your patients caused by wearing the ambulatory blood pressure monitor for 24 hours? (Adverse effects)*

1. Were there any benefits, profits, or values you had to give up in order to perform ABPM? (**Opportunity cost**)

*Probes:*

- 1. *What are your thoughts about the cost of using ABPM? (Cost)*
  2. *Looking back on your previous experiences with performing ABPM, do you feel that it ever offset your other clinical responsibilities or tasks? If so, can you describe how you managed to balance your workload?*
  3. *Did you encounter any other challenges or concerns when performing ABPM?*

**Questions on using ABPM in the clinic/facility**

1. Based on your experience of using ABPM, what are your thoughts on using it in your organization?

*Probes:*

- 1. *How well do you think using ABPM will meet the needs of your patients? (Patient needs and resources)*
  2. *Is there a strong need to change the current blood pressure measurement approach? Or is there an alternative approach that has been considered for the blood pressure measurement? (Tension for change)*

1. How well do you think ABPM will fit into your organization? Why or why not?

*Probes:*

- 1. *Do you feel your organization is well-equipped to adopt ABPM for managing hypertension? (Structural characteristics)*
  2. *How will the use of ABPM fit with the current services and supports in your organization? What are the likely issues or problems that may arise? (Compatibility)*
  3. *What do you think will be the changes or modifications to the ABPM process to make it work effectively here? (Adaptability)*

1. What kind of support or resources do you think is needed to make things easier for you to use ABPM in your organization?
   1. *How ready do you think your organization is to adopt ABPM? For example, what resources are required to adopt ABPM in your organization? (Readiness to change)*
2. Do you have anything else you would like to share on this topic?

That's the end of our interview. Thank you for taking your time with me today. [Turn off the recorder]

**Interview Guide for Healthcare Administrators**

Thank you for agreeing to take part in this study. Today, we are hoping to learn about your acceptability of using ambulatory blood pressure monitoring (ABPM) for blood pressure monitoring in the clinic/facility and its potential influencing factors. Before we begin, I’d like to explain a few things about the study and how the interview will work.

Firstly, this interview will take around 30-60 minutes. The conversation will be recorded and transcribed, but no confidential information will be mentioned. As a reminder, your participation in this study is voluntary. You may choose to not answer some of the questions or end the interview at any time. If you are unsure about anything we ask, please feel free to ask for clarification or ask us to repeat the question.

Secondly, we understand that there may be some confusion or misunderstanding about the device, particularly if you have experience using other approaches for blood pressure monitoring. Therefore, I would like to provide you with some information and show you some pictures of the device we will be discussing today. Ambulatory blood pressure monitoring, or ABPM, is a medical device that is used to measure your blood pressure at regular intervals over a 24-hour period. Here are some photos of what the device looks like [show photos].

Before we begin, do you have any questions about what I have just explained? [answer questions]

May I start recording the interview? (After gaining permission, switch on the recorder) Ok, thanks for letting us record this.

**Opening questions**

1. To start with, would you tell me something about your current position at [organization]?

*Probes:*

1. *What is your current position/title?*
2. *How long have you worked here?*

**Questions on Experienced Acceptability**

1. Please share with me your experiences in performing ambulatory blood pressure monitoring (ABPM) tests.

*Probes:*

1. *How often did you perform ABPM test in your organization?*
2. *Could you describe the process of ordering, administering, and interpreting ABPM tests in your organization and what was your role in it?*
3. What are your thoughts on ABPM?

*Probes:*

- 1. *What did you like or dislike about using ABPM in your organization?* ***(Affective attitude****)*
  2. *How well did the use of ABPM fit with your organization’s ethical values and principles? (****Ethicality****)*
  3. *Compared to other methods of blood pressure measurement, what features or characteristics did ABPM have that stood out to you? (Relative advantage)*

1. How convinced were you about the effectiveness of ABPM? (**Perceived Effectiveness**)

*Probes:*

- 1. *How did ABPM compare to other methods of blood pressure measurement in terms of accuracy and patient compliance?*
  2. *What role did you believe ABPM play in the overall management of hypertension?*

1. How confident were you that clinicians would be able to successfully perform ABPM in your organization? (**Self-efficacy**)

*Probes:*

- 1. *What steps did your organization take to ensure your clinicians were properly prepared to perform ABPM?*

1. On a scale of 1-10, how would you rate your clinicians’ understanding of how ABPM works in your organization? Would you elaborate on this? (**Intervention Coherence**)

*Probes:*

- 1. *How did you ensure that the results of ABPM were properly interpreted and integrated into patient care plans?*

1. How much effort did your organization take to perform ABPM? (**Burden**)

*Probes:*

- 1. *Were there any particular challenges or obstacles to perform ABPM in your organization, such as staffing, training, or equipment availability?*
  2. *What were your thoughts on the possible adverse effects for your patients caused by wearing the ambulatory blood pressure monitor for 24 hours? (Adverse effects)*

1. What benefits, profits, or values did you give up to perform ABPM in your organization? (**Opportunity cost**)

*Probes:*

- 1. *What are your thoughts about the cost of using ABPM? (Cost)*
  2. *Did you have any other challenges or concerns about performing ABPM in your organization?*

**Questions on Using ABPM in the clinic/facility**

1. Based on your experience of performing ABPM, what are your thoughts on using it in your organization?

*Probes:*

- 1. *How well do you think using ABPM will meet the needs of your patients? (Patient needs and resources)*
  2. *Do you think there's a strong need to change the current blood pressure measurement approach? Why or why not? (Tension for change)*
  3. *To what extent will using ABPM in your organization take a backseat to other high-priority initiatives? (Relative Priority)*

1. Do you believe using ABPM will be effective in your organization? Why or why not?

*Probes:*

- 1. *Does your organization have the infrastructure to support the use of ABPM? (Structural characteristics)*
  2. *How will the use of ABPM fit with the current services and supports in your organization? (compatibility)*
  3. *What do you think will be the changes or modifications to the ABPM process to make it work effectively here? (Adaptability)*

1. What kind of support do you think is needed to make things easier for you to use ABPM in your organization?
   1. *How ready do you think your organization is to adopt ABPM? For example, do you expect to have sufficient resources to adopt ABPM in your organization? (Readiness to change)*
2. Do you have anything else that you would like to share on this topic?

That's the end of our interview. Thank you for taking your time with me today. [Turn off the recorder]
