# Supplementary material for: Identifying barriers to the acceptability and adoption of ambulatory blood pressure monitoring and proposed strategies in Bangladesh, Pakistan, and Sri Lanka: a qualitative study
Source: BMC Health Serv Res. 2026 Feb 3;26:237. doi: 10.1186/s12913-026-14107-y (PMC12903567; doi:10.1186/s12913-026-14107-y)
Supplement: Supplementary file 4 — Supplementary Material 4 [file 12913_2026_14107_MOESM4_ESM.docx]

**Supplementary File 4 – Analysis Codebook**

| **Codes** | **Original Definitions** | **Adjusted Definitions** | **References** |
| --- | --- | --- | --- |
| **The Theoretical Framework of Acceptability (TFA)** | | | |
| Affective attitude | The overall positive or negative feeling towards the intervention. | The feelings and attitudes of healthcare professionals and patients with hypertension towards ABPM adoption | TFA-based |
| Patients' anxiety of taking ABPM |  | Anxiety or stress experienced by patients due to ABPM, particularly concerning its operation or the sensation during measurement. | Open-coded |
| Patients’ future plan to use ABPM |  | The extent to which patients with hypertension intend to incorporate ABPM into their future hypertension management, including their perceived need and willingness. This also includes healthcare professionals' view on the cost-effectiveness of ABPM, considering upfront costs with long-term benefits, such as better justification for lifelong medications. | Open-coded |
| Ethicality | The extent to which the intervention aligns with the participant’s value system. | How well the use of ABPM aligns with ethical considerations of patients with hypertension and healthcare professionals.  For healthcare professionals, this includes ensuring informed patient consent, data security, equity, clinical justification (e.g. avoiding unnecessary treatment for individuals with white coat hypertension), and considering any potential negative effects.  For patients, this includes perceptions of equity, willingness to recommend ABPM to others, and an overall sense of respect and fair treatment throughout the process. | TFA-based |
| Self-efficacy | The participant’s confidence in their ability to perform the intervention. | Confidence of healthcare professionals in their ability to effectively apply, interpret and manage ABPM, addressing potential challenges.  Confidence of patients with hypertension in their ability to effectively use ABPM. | TFA-based |
| Perceived effectiveness | The extent to which the intervention is perceived as likely to achieve its purpose. | The perceived effectiveness of patients with hypertension and healthcare professionals on ABPM in providing accurate and useful data for hypertension management. | TFA-based |
| Perceived accuracy of ABPM in daily life |  | The belief that ABPM is most effective when conducted in a patient’s natural environment, allowing for accurate reflection of blood pressure fluctuations as they occur in daily life activities. | Open-coded |
| Burden | The perceived amount of effort required to participate in the intervention. | The perceived extent of effort and strain experienced by healthcare professionals and healthcare staff when integrating ABPM into their clinical routines.  The perceived inconvenience, discomfort, and disruption experienced by patients using ABPM, including physical, social, and psychological aspects. | TFA-based |
| User Discomfort |  | Potential skin discomforts, pain, and sleep disruptions patients might experience from using ABPM | Open-coded |
| Appointment and return challenges |  | Challenges in scheduling and managing ABPM appointments, including organizational concerns about device non-return and patient issues such as waiting times, travel distances, and any other logistical differences. | Open-coded |
| Patient adherence conditions |  | Instances or risks of patients either following through with ABPM procedures and adhering to device usage guidelines or failing to do so. Non-adherence can cause a burden on healthcare professionals due to inaccurate results and pose risks to patients. | Open-coded |
| Opportunity cost | The extent to which benefits, profits, or values must be given up to engage in the intervention. | The potential losses healthcare professionals might incur due to the adoption of ABPM, such as time that could have been spent on other patient care activities or administrative duties.  The trade-offs that patients face when choosing to use ABPM over other activities or treatments. It includes the time spent managing and wearing the device instead of engaging in other valuable activities, as well as the potential income loss due to medical appointments or reduced ability to work. | TFA-based |
| Disturbing patients' daily life |  | How ABPM affects patients' daily activities and quality of life, including sleep, religious activities (e.g. prayers), or routine tasks such as shopping or taking a bath. | Open-coded |
| Intervention coherence | The participant’s understanding of the intervention and how it works. | How well healthcare professionals understand the use and benefits of ABPM, reflecting their grasp of its purpose and functionality.  The degree to which patients are informed about ABPM, including its purposes, usage, expected results, and what to expect during monitoring. | TFA-based |
| **The Consolidated Framework of Implementation Research (CFIR)** | | | |
| **ABPM usage characteristics** | | | |
| Applicable population and conditions |  | The group of individuals and the types of medical conditions that healthcare professionals and patients believe would benefit most from ABPM. This also includes the perspective that ABPM may not be suitable or beneficial for all patients, requiring consideration of its appropriate use. | Open-coded |
| Cost of testing | Costs associated with the intervention including initial costs, ongoing costs, and opportunity costs. | The financial impact of ABPM on patients and the organization, including considerations of affordability. This includes patients’ willingness and capability to pay for ABPM, as well as healthcare professionals’ considerations of the financial implications when referring ABPM. | CFIR-based |
| Evidence strength and quality | Reflects stakeholders' perceptions of the credibility and strength of evidence supporting the intervention’s effectiveness. | Empirically supported benefits of using ABPM, based on research and clinical studies. | CFIR-based |
| Device size, weight, design, and quality | The perceived excellence in how the intervention is bundled, presented, and assembled. (Design quality and packaging) | Physical attributes of the ABPM device, such as its size, weight, quality, and ease of use. | CFIR-based |
| Procedure complexity | The perceived difficulty of the intervention, reflected by duration, scope, radicalness, disruptiveness, centrality, and intricacy and number of steps required to implement. | Perceived complexity of the ABPM procedure from the appointment to returning the device. | CFIR-based |
| Relative advantage | The stakeholder's perception of the advantages of implementing the intervention versus an alternative solution. | The perceived benefits of ABPM over other blood pressure monitoring methods in terms of accuracy, convenience, and data reliability. | CFIR-based |
| **Inner setting** |  |  |  |
| Organizational readiness for ABPM | Tangible and immediate indicators of organizational commitment to its decision to implement an intervention. | The readiness of the current organization to implement ABPM effectively, considering available resources and training. | Adapted from CFIR |
| Available resources | The level of resources dedicated for implementation and ongoing operations, including money, training, education, physical space, and time. | The availability of ABPM devices, staffing, time, and financial resources necessary to ensure proper ABPM usage and management within the organizations, including whether there are enough devices for patient needs. | Adapted from CFIR |
| Access to knowledge & information | Ease of access to digestible information and knowledge about the intervention and how to incorporate it into work tasks. | Ease of access to and availability of training materials and guidelines for the correct use of ABPM for both patients and healthcare professionals. | Adapted from CFIR |
| Tension for change | The degree to which stakeholders perceive the current situation as intolerable or needing change. | The extent to which healthcare professionals and patients with hypertension perceive current ABPM practices as either insufficient or inadequate, leading to a strong desire for change. This includes calling for increased use in specific situations or recognizing gaps in adherence to clinical guidelines. | Adapted from CFIR |
| Compatibility with Organization | The degree of tangible fit between meaning and values attached to the intervention by involved individuals, how those align with individuals’ own norms, values, and perceived risks and needs, and how the intervention fits with existing workflows and systems. | How well the use of ABPM fits into healthcare professionals' regular workflow and duties, as well as its alignment with the broader mission and priorities of the organization in managing hypertension, including improving patient outcomes and adhering to evidence-based guidelines. | Adapted from CFIR |
| Relative priority | Individuals’ shared perception of the importance of the implementation within the organization. | The shared perception among healthcare professionals and managers regarding the importance and urgency of adopting ABPM within their healthcare setting. This includes how ABPM is prioritized compared to other medical technologies and procedures for hypertension management in terms of resources, attention, and effort. | Adapted from CFIR |
| Fulfillment of diagnostic needs |  | Whether ABPM is seen as meeting the diagnostic and monitoring needs of patients effectively. | Open-coded |
| Importance of patient counseling |  | The role and impact of patient counseling in the ABPM test procedure, such as its effectiveness in easing patient anxiety, ensuring correct usage, and persuading patients to undergo the test due to its benefits. | Open-coded |
| Adherence to guidelines among healthcare professionals |  | The extent to which healthcare professionals follow established guidelines and protocols for BP-related activities, such as BP measurement, prescribing ABPM tests, and prescribing antihypertensive medications, as well as the reasons for adherence or non-adherence. | Open-coded |
| Trust in organizational processes and quality of care |  | The level of trust healthcare professionals have in the reliability of organizational processes and the overall quality of care, which affects their confidence in adopting ABPM. This includes whether they believe their organizations would follow guidelines that support ABPM use and maintain high standards of patient care. | Open-coded |
| Disparities in ABPM resources and provision across healthcare settings |  | The differences in availability and quality of ABPM devices, related workforce, and provision of ABPM tests between private and public healthcare settings. | Open-coded |
| Concern of not returning or mishandling ABPM devices |  | Challenges faced by healthcare professionals regarding the risk of patients not returning or mishandling ABPM devices. This includes accountability issues and differences in device management between public and private healthcare settings, such as the use of deposits, patient trust, and institutional safeguards to mitigate potential financial and logistical losses. | Open-coded |
| Device procurement and acquisition |  | The processes by which ABPM devices are obtained within healthcare settings, including the pathways (e.g. purchases, donations) and operational steps involved in bringing the devices into use. | Open-coded |
| **Outer setting** |  |  |  |
| External policies and incentives | A broad construct that includes external strategies to spread interventions, including policy and regulations (governmental or other central entity), external mandates, recommendations and guidelines, pay-for-performance, collaboratives, and public or benchmark reporting. | Economic (e.g., healthcare insurance coverage, subsidies), political (e.g., national or regional guidelines that encourage or regulate the use of ABPM), and/or technological conditions (e.g., availability of ABPM devices in the market, supporting infrastructure) that enable healthcare organizations to support the adoption of ABPM. | Adapted from CFIR |
| Social stigma about ABPM users |  | Negative perceptions and reactions from the community and others towards patients using ABPM, affecting patient comfort and acceptance. | Open-coded |
| Expansion of ABPM adoption |  | Views on the potential for broader implementation of ABPM in other healthcare settings or contexts. | Open-coded |
| Local awareness of ABPM benefits |  | The extent to which the benefits of ABPM are known and understood within the local community, including patients, healthcare professionals, policy makers, and the general public. This includes the level of awareness or lack thereof of ABPM, along with the recognition that education on ABPM could improve its adoption and usage. | Open-coded |
| Inequitable access to ABPM tests |  | Patient Perspective: Barriers faced by individual patients in accessing and utilizing ABPM tests, such as socio-economic status, geographic location, and healthcare coverage.  Healthcare professional/Organization Perspective: Barriers faced by healthcare professionals and organizations in offering ABPM tests, such as limited fundings, availability of devices, and necessary resources, highlighting disparities in resource allocation. | Open-coded |

Abbreviations: ABPM, Ambulatory Blood Pressure Monitoring; CFIR, The Consolidated Framework for Implementation Research; TFA, Theoretical Framework of Acceptability.
