# Supplementary material for: Identifying barriers to the acceptability and adoption of ambulatory blood pressure monitoring and proposed strategies in Bangladesh, Pakistan, and Sri Lanka: a qualitative study
Source: BMC Health Serv Res. 2026 Feb 3;26:237. doi: 10.1186/s12913-026-14107-y (PMC12903567; doi:10.1186/s12913-026-14107-y)
Supplement: Supplementary file 5 — Supplementary Material 5 [file 12913_2026_14107_MOESM5_ESM.docx]

# Supplementary File 5 – Author Reflexivity Statement

This consensus statement is specifically designed to address research equity within international partnerships. This is an issue of major priority in low- and middle-income countries (LMIC) whose researchers engage in collaboration with high-income country researchers.

## How does this study address local research and policy priorities?

Our research aimed to explore barriers and proposed strategies for the acceptability and adoption of ambulatory blood pressure monitoring (ABPM) in South Asia. This was motivated by the region’s high burden of hypertension and the observed underutilization of ABPM despite international guideline recommendations. We believed understanding local perspectives would inform more effective hypertension management.

## How were local researchers involved in study design?

This study was co-developed by a multidisciplinary, multinational team, including researchers from Singapore (THJ, AZ, SY), Pakistan (AA, MSK), Bangladesh (AN, NC, NF), and Sri Lanka (HAdS, CKdS, LA). The initial interview guide was developed by the Singapore team based on prior studies and the combined framework of the Theoretical Framework of Acceptability (TFA) and the Consolidated Framework of Implementation Research framework (CFIR). Researchers from each South Asian country then reviewed and discussed the interview guides, suggesting culturally relevant additions and modifications based on their contextual experience, such as specific questions on religious practices during ABPM use.

## How has funding been used to support the local research team?

This study has allocated fundings to pay salaries of data collectors and analysts in each country. It also covered the annual NVivo 14 license fees for all teams to enable qualitative data analysis, provided facilities and equipment such as voice recorders, stationary, and consumables, and supported local transportation fees for field work. Reimbursement for research participants was also included in the study budget. The study was funded by the SingHealth Duke-NUS Global Health Institute (SDGHI) under Award Number SDGHI_PGA_FY2022-03.

## How are research staff who conducted data collection acknowledged?

Local researchers responsible for data collection and analysis in each country are acknowledged. Researchers responsible for data collection and analysis who did not have substantive input into study design or manuscript writing are acknowledged by name in the Acknowledgements section. Principal investigators (PI) and site coordinators are included as co-authors based on their substantial contributions, in line with ICMJE authorship criteria.

## Do all members of the research partnership have access to study data?

Due to institutional review boards (IRB) requirements, only de-identified questionnaires and transcripts were shared with the Singapore team. The research team in Bangladesh, Pakistan, and Sri Lanka had full access to the data collected from their own sites, while ensuring participant confidentiality was maintained throughout the study.

## How was data used to develop analytical skills within the partnership?

In this study, data analysis workshops were conducted to build local researchers’ skills in qualitative analysis. These covered the use of NVivo 14 for line-by-line coding, principles of framework analysis, and the application of both deductive and inductive coding. Providing NVivo licenses and involving local researchers in codebook development and data interpretation further supported their capacity development in this area.

## How have research partners collaborated in interpreting study data?

Data analysis was jointly conducted: the primary codebook was developed by the Singapore team, after which local researchers independently analysed transcripts from their own contexts and updated the codebook in collaboration with AZ, ensuring that cultural sensitivity was incorporated.

## How were research partners supported to develop writing skills?

The initial manuscript draft was prepared by AZ, with feedback and guidance from THJ and SY. This draft was then circulated to all research partners, who were invited to provide feedback, contribute revisions, and approve final content. This collaborative process ensured that all co-authors were engaged in shaping the manuscript.

## How will research products be shared to address local needs?

This study will be published as open access. We also plan to disseminate our findings to local stakeholders (e.g., local healthcare institutions, policymakers, and community organizations) through summary reports and presentations in local languages.

## How is the leadership, contribution and ownership of this work by LMIC researchers recognised within the authorship?

Eight LMIC researchers who made substantive contributions to the study’s design, data collection, analysis, and interpretation are listed as coauthors (AA, AN, HAdS, CKdS, LA, NC, MSK, and NF). We acknowledge that the first and corresponding authors are currently based in Singapore, a high-income country. However, authorship order was determined by overall contributions across all stages of the research, in line with ICMJE guidelines, to ensure appropriate recognition of leadership and ownership by LMIC researchers.

## How have early career researchers across the partnership been included within the authorship team?

We have included early career researchers (AZ, CKdS, NC, and MSK) within the authorship team. AZ, as the first author, was actively involved in study design, data analysis, and manuscript preparation as part of her Ph.D. project. CKdS, NC, and MSK contributed to both data collection and analysis.

## How has gender balance been addressed within the authorship?

Eight authors identified as female (AZ, SY, AA, AN, CKdS, LA, NF, and TJ) and three authors male (HAdS, NC, and MSK).

## How has the project contributed to training of LMIC researchers?

The project provided hands-on training in qualitative research methods, including data collection, management, and analysis through three workshops.

Recordings of the three workshops and relevant educational materials were also shared across the three countries for future educational purpose, if needed.

## How has the project contributed to improvements in local infrastructure?

This project has not directly contributed to improvements in local infrastructure.

## What safeguarding procedures were used to protect local study participants and researchers?

Ethical approval was obtained from all relevant IRB, and informed consent was secured from all participants. Data were anonymized to protect participant confidentiality, and researchers received training in confidentiality and safety protocols. De-identified data were stored and shared via a password-protected cloud storage platform, in accordance with IDPPA and National University of Singapore data protection requirements.
